# Welfare Risks of Repeated Application of On-Farm Killing Methods for Poultry

**DOI:** 10.3390/ani8030039

**Published:** 2018-03-15

**Authors:** Jessica E. Martin, Dale A. Sandercock, Victoria Sandilands, Julian Sparrey, Laurence Baker, Nick H. C. Sparks, Dorothy E. F. McKeegan

**Affiliations:** 1 The Royal (Dick) School of Veterinary Studies and The Roslin Institute, Easter Bush Campus, The University of Edinburgh, Edinburgh EH25 9RG, UK; Nick.Sparks@roslin.ed.ac.uk; 2 Animal and Veterinary Science Research Group, Scotland’s Rural College (SRUC), West Mains Road, Edinburgh EH16 4SA, UK; dale.sandercock@sruc.ac.uk (D.A.S.); Vicky.Sandilands@sruc.ac.uk (V.S.); laurence.baker@sruc.ac.uk (L.B.); 3 Livetec Systems Ltd, Building 52, Wrest Park, Silsoe, Bedford MK45 4HS, UK; sparrey@livetecsystems.co.uk; 4 Institute of Biodiversity, Animal Health and Comparative Medicine, College of Medical, Veterinary & Life Sciences, University of Glasgow, Glasgow G61 1QH, UK; Dorothy.McKeegan@glasgow.ac.uk

**Keywords:** killing, poultry, cervical dislocation, captive bolt, reflexes, animal welfare.

## Abstract

**Simple Summary:**

During poultry production, some birds are killed humanely on farm, usually because they are ill or injured. Recent European Union (EU) legislation has restricted the number of birds that can be killed by manual neck dislocation to 70 birds per person per day. We examined whether this limit is meaningful by investigating the effects of repeated application of two methods of killing (neck dislocation and a percussive method, the CashPoultry Killer). Twelve male stockworkers each killed 100 birds (broilers, laying hens, or turkeys) at a fixed rate with each method. Both methods were highly successful, and reflex and behaviour measures confirmed they caused rapid loss of brain function. Importantly, there was no evidence of reduced performance with time/bird number up to 100 birds with either method. The Cash Poultry Killer caused a more rapid death, but it was prone to technical difficulties with repeated use. Neck dislocation has the important advantage that it can be performed immediately with no equipment, which may make it preferable in some situations. We present the first evidence that, at the killing rates tested, there was no evidence to justify the current EU number limit for performance of neck dislocation to kill poultry on farm.

**Abstract:**

Council Regulation (EC) no. 1099/2009 on the protection of animals at the time of killing restricts the use of manual cervical dislocation in poultry on farms in the European Union (EU) to birds weighing up to 3 kg and 70 birds per person per day. However, few studies have examined whether repeated application of manual cervical dislocation has welfare implications and whether these are dependent on individual operator skill or susceptibility to fatigue. We investigated the effects of repeated application (100 birds at a fixed killing rate of 1 bird per 2 min) and multiple operators on two methods of killing of broilers, laying hens, and turkeys in commercial settings. We compared the efficacy and welfare impact of repeated application of cervical dislocation and a percussive killer (Cash Poultry Killer, CPK), using 12 male stockworkers on three farms (one farm per bird type). Both methods achieved over 96% kill success at the first attempt. The killing methods were equally effective for each bird type and there was no evidence of reduced performance with time and/or bird number. Both methods of killing caused a rapid loss of reflexes, indicating loss of brain function. There was more variation in reflex durations and post-mortem damage in birds killed by cervical dislocation than that found using CPK. High neck dislocation was associated with improved kill success and more rapid loss of reflexes. The CPK caused damage to multiple brain areas with little variation. Overall, the CPK was associated with faster abolition of reflexes, with fewer birds exhibiting them at all, suggestive of better welfare outcomes. However, technical difficulties with the CPK highlighted the advantages of cervical dislocation, which can be performed immediately with no equipment. At the killing rates tested, we did not find evidence to justify the current EU limit on the number of birds that one operator can kill on–farm by manual cervical dislocation.

## 1. Introduction

Poultry may need to be dispatched on farm for multiple reasons such as culling of individual injured and sick birds, and for stock management. The methods used to cull small numbers of birds on farm are different than those for slaughter [1,2,3] or emergency on-farm killing for disease control [4,5], relying heavily on manual cervical dislocation (so called ‘necking’ by hand) [6]. Manual cervical dislocation has been a source of welfare concern for poultry [7,8,9] and other species [10,11], and as of January 2013, the use of this method to kill poultry on-farm has been restricted to birds weighing a maximum of 3 kg and 70 birds per person per day through European Union (EU) legislation, Council Regulation (EC) no. 1099/2009 on the protection of animals at the time of killing [12]. These restrictions were imposed primarily in response to concern that animals may be conscious for a significant period post-application of cervical dislocation [7,13,14], since the method is thought to kill birds primarily by cerebral ischemia which is not instantaneous [7,9,13,14]. However, recent work suggests that manual cervical dislocation may be more humane than previously thought [9,15,16,17], and it may be that the results of earlier studies did not correctly reflect the efficacy of manual cervical dislocation when done correctly or instead assessed mechanical cervical dislocation (i.e., use of a tool for dislocation) [18]. It has also been noted that there is variability in the application of any type of cervical dislocation method (manual or mechanically-assisted) by different operators (e.g., stockworkers, veterinarians, trained slaughtermen) for different poultry species [6,17,19], making generalizations problematic.

The uptake of killing methods for small numbers of poultry depends on their practicality, cost, and availability for rapid deployment, because the majority of emergency killing needs to be done immediately and on farm sites. Manual cervical dislocation is attractive because it requires no equipment and can be performed anywhere. Although mechanically-assisted cervical dislocation shares many of these advantages, it does require a tool or aid (e.g., killing cone, pliers [6,18]). Alternative killing methods involve destruction of the brain primarily via application of captive bolt or percussion, and several of these methods have been developed over the last ten years (e.g., Cash Poultry Killer (CPK), Turkey Euthanasia Device) [7,14,18,20,21,22,23]. Several studies have concluded that the captive bolt renders birds unconscious immediately or rapidly post application, inferring that this is a humane method of killing [7,24,25]. 

The welfare implications of both percussive and cervical dislocation techniques are reliant on skilful application by a human operator, with human error potentially having major welfare consequences. Variation in application of cervical dislocation (manual or mechanically-assisted) has been previously documented [17] and demonstrates a lack of standardization in training in the poultry industry. However, there is very little research investigating the welfare implications of repeated application of on-farm killing methods. One study investigated the performance of manual cervical dislocation in up to 60 birds in succession, and showed that while efficacy did not change over time, there was substantial variation between stockworkers [17]. There is no such study for captive bolt methods for poultry. Therefore, the evidence basis for legal restrictions on manual cervical dislocation is unclear, and there is a need to examine the effects of repeated performance (i.e., risk of fatigue affecting welfare outcomes) up to and exceeding this limit for both cervical dislocation and percussive methods.

The aim of this study was to assess the effects of repeated application (up to 100 birds) and multiple operators on the killing efficacy and welfare impact of two commercially relevant killing methods: the CPK [18,23] and cervical dislocation. Two forms of cervical dislocation were assessed: manual (broilers and layers) and mechanically-assisted (turkeys).

## 2. Materials and Methods

A total of 2400 female birds were used; 800 turkey hens (25 weeks old, Kelly’s Bronze, *Meleagris gallopavo*), 800 layer hens (76 weeks old, HyLine Brown, *Gallus gallus domesticus*), and 800 broilers (30-32 days old, Ross 308, *Gallus gallus domesticus).* The study was conducted in collaboration with three commercial poultry producers, on their farms. All birds sourced for the experiment were either destined to go to slaughter (broilers and turkeys) or were being killed at the end of their productive life (end-of-lay hens). The birds were reared and housed commercially until killing occurred. The layer hens were housed in enriched colony cages (Tecno Cages^®^, Tecno Poultry Equipment Spa, EU) with 80 birds per colony. The broilers and turkeys were floor housed in a clear span shed with deep litter (wood-shavings). All birds had ad libitum access to food and water. The experiment was performed under the United Kingdom’s (UK’s) Animal Scientific Procedures Act, and as part of this, underwent review and approval by SRUC’s AWERB (AU AE 49-2012, 22 November 2012).

**Figure 1:**
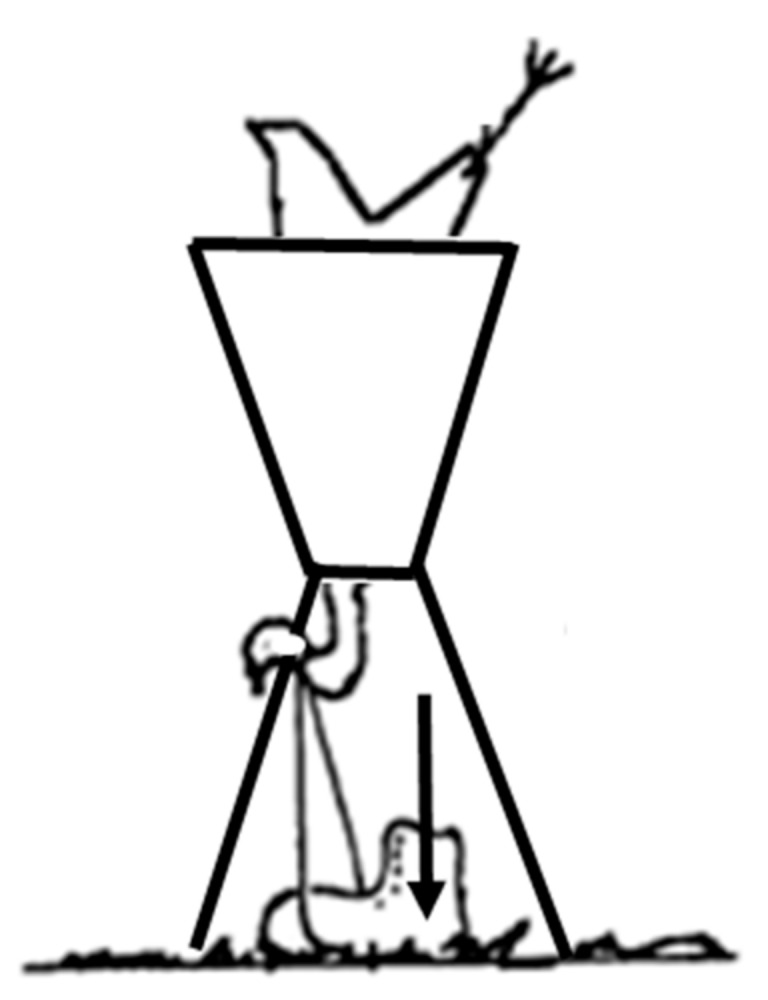
Mechanically-assisted cervical dislocation for turkeys. The bird is restrained in a cone (inverted position). A nylon cord is looped (600 mm) over and behind the head at the cranial/vertebral (C0/C1) junction. Holding the head firmly in place (by hand), the stockworker places his foot through the lower part of the loop situated beneath the bird (off the ground) and forcefully and quickly presses his foot to the floor causing a rapid dislocation of the neck.

The experiment was a balanced factorial design: four stockworkers × two kill methods × 100 birds × three bird types in order to assess the repeated performance of cervical dislocation and CPK for operator consistency of method application (e.g., potential fatigue). Two types of cervical dislocation were used: manual cervical dislocation for broilers and layer hens and mechanically-assisted cervical dislocation for turkeys, which mimicked commercial practice and conformed to the EU Directive (EU 1099/2009). The manual cervical dislocation used was dependent on the stockworker’s previous training and standard operating procedures at each farm, and did not always follow Humane Slaughter Association’s (HSA’s) guidelines [18]. Variation in the techniques related to how the bird’s head was held in the operator’s palm [17]. Manual cervical dislocation was performed in one swift movement with the operator pulling down on the bird’s head, stretching the neck, while rotating the bird’s head upwards into the back of the neck, as detailed in [16,17,18]. Mechanically-assisted cervical dislocation involved restraining and inverting the bird within a cone. The stockworker held the bird’s head in one hand and placed a nylon cord loop (~60 cm) over and behind the head at the cranial/vertebral (C0/C1) junction on the neck. Holding the head firmly in place, the stockworker placed his foot through the lower part of the loop situated beneath the bird and forcefully and quickly pressed his foot to the floor causing a rapid dislocation of the neck (Figure 1).

The second killing method was the Accles and Shelvoke Cash Poultry Killer .22 CPK 200, (using a 1 grain (65 mg) gunpowder cartridge), a non-penetrating captive bolt percussive device. To apply the CPK, the bird’s body was held in sternal recumbency by an assistant stockworker, with the bird’s head hanging freely. The beak tip was held by the stockworker applying the CPK, ensuring that free space was available for the head to swing away. The CPK was aimed at the top of the head, with the lower curved edge of the cowl midway between the eyes, with the device slightly angled towards the tail (following instructions in the manufacturer’s [23] and Humane Slaughter Association (HSA) guidelines [18]).

Twelve male stockworkers (four per farm) volunteered for the trial and were pre-screened for experience in performing cervical dislocation and were deemed competent by their respective managers. All stockworkers completed a full training day in the CPK, provided and audited by the HSA in November 2012, and all were deemed competent by the HSA representative. Biometric data for each stockworker was recorded on site and included: age (years), height (m), weight (kg), arm length (cm), and hand span (cm). The stockworkers were also asked to provide a rough estimate of how many years of experience they had in performing cervical dislocation as an emergency killing method.

On each farm, 100 birds were killed with each method (cervical dislocation and CPK) per stockworker. For commercial logistical reasons, the culling sessions for all four stockworkers per farm were done over a four day period. Each stockworker performed one killing method per day. The kill method order, day, time of day, and the stockman’s role (assist or kill) was allocated and balanced according to a Latin square design framework within each farm. On each farm, the four stockworkers were paired, where one acted as the assistant to the other (handling/catching birds), and the other conducted the killing (100 birds per session). After the completion of one session, the stockworkers swapped roles within their pairs, with a break between sessions. Within each kill session, the stockworkers were not permitted to have a break, however, a predetermined killing rate of one bird per 2 min was set in order to provide enough time for the recording of reflex and post-mortem measures for every 10th bird, to prevent kill rate becoming a confounding factor between individuals and to allow comparisons between stockworkers to be made accurately across farm and bird types. Fatigue could not be measured directly within stockworker, but was implied indirectly by kill sequence (1–100) per stockworker per session. All birds were weighed and identified with a numbered leg tag immediately prior to killing. Each killing session took approximately 3.5 h to complete without any breaks.

Death was confirmed in every bird by an experienced poultry technician immediately post application of a method by assessment of two parameters: (1) cessation of rhythmic breathing and (2) absence of pupillary reflex [4,26]. The same technician recorded the number of kill attempts (e.g., multiple shots or cervical dislocation pulls) and assessed and recorded kill efficacy, which was defined as a single kill attempt resulting in rapid death and no emergency intervention required. If any bird did not display signs of death rapidly post-application, they were immediately emergency euthanized with a back-up CPK device operated by the poultry technician.

A gross post-mortem examination was performed on every bird immediately after the application of the killing method and confirmation of death by a trained poultry technician. General binary yes/no post-mortem measures were recorded for all birds: skin broken, external blood loss, and subcutaneous hematoma. Kill method specific post-mortem measures were obtained with regard to damage to specific anatomical areas related to target areas of the approaches. For cervical dislocation methods, four post-mortem parameters were recorded: cervical dislocation confirmation (y/n), the level of vertebral dislocation (e.g., C0–C1), spinal cord severed (y/n), and the number of carotid arteries severed (0, 1, or 2). For the CPK method, seven gross post-mortem parameters were recorded: skull fracture (y/n), skull fracture location [16], damage (y/n) to the forebrain (left and right), midbrain (left and right), and cerebellum [15,16,17]. In order to establish gross anatomical damage, the brain was excised in order to accurately visualize each brain region. Any birds which underwent emergency euthanasia as a result of a failed kill were excluded from post-mortem data since their anatomical damage was confounded by the emergency method of killing. Based on post-mortem measures for any birds which were successfully killed (as defined above), if the optimal anatomical damage was achieved (e.g., cervical dislocation = C0–C1, both carotid arteries and spinal cord severed, and no skin broken; CPK = profuse damage to >1 brain region), the kill was classified as a ‘method success’.

For the first and every tenth bird within a session (total of 11 birds), reflex and behaviour latencies were recorded immediately post method application. Three cranial reflexes (pupillary [27], nictitating membrane [7,28], and rhythmic breathing [7,29]) and four relevant involuntary behaviours (presence of jaw tone [4,26], cloacal movement [4], and clonic wing flapping and leg paddling [4,7,26]) were assessed as present or absent at 15 s intervals post killing treatment application until an uninterrupted 30 s of absence of all behaviours and reflexes was observed. Descriptions of the reflexes and behaviours and methods of assessment have been validated in previous studies [7,16,26]. Measures were recorded in a predetermined order for each observer, and using the 1-0 sampling technique [30]: if a reflex/behaviour was present during any point of a 15 s interval, it was defined as present for the entire interval, providing a conservative measure of reflex/behaviour duration post killing treatment application. This interval count was placed on the time scale by assuming counts 0, 1, 2, 3…, to be 3, 10, 20, 35…etc., the midpoint of each reflex interval. The justification for no observation being conducted prior to 3 s post method application was that it was assumed that the reflex measurement could not be undertaken prior to this due to logistical reasons (e.g., bird hand over). If a reflex or behaviour could not be recorded, for example, if pupillary reflex was concealed due to damage to the eye, the data was recorded as missing.

### Statistical Analysis

Data were collected at the bird level and stockworker level and were summarized in Microsoft Excel (2010) spreadsheets and analysed using Genstat (16th Edition). Statistical significance was termed by a threshold of 5% probability based on F tests. Summary graphs and statistics were produced at the stockworker level. For all models, the random effects included the stockworker. All fixed effects were treated as factors and classed as categorical classifications.

Generalized Linear Mixed Models (GLMMs) using logit link function and binomially distributed errors due to the nature of the binary data were used to statistically compare kill efficacy and post-mortem parameters across stockworkers. Dispersion was fixed dependent on the variable. Random effects included in models were day of kill (*n* = 12) and kill session (*n* = 24), and stockworker (*n* = 12). In the maximal models, fixed effects included killing method, bird type, bird order, session, and all their interactions. Co-variates included bird weight and number of years of stockworker experience.

For the subset of birds subject to reflex measurements (*n* = 264 birds), the presence/absence of each reflex and behaviour was summarized into interval counts (e.g., present in 0–15 s = 1 count), therefore, the data was summarized into means of the maximum interval counts at the bird level for each reflex, which were then converted back into the time dimension(s). GLMMs with logit-link function and Poisson distributed errors were fitted to the interval counts. Overall statistical comparisons across the killing treatments were conducted. Random effects included in models were day of kill, kill session, and stockworker. In the maximal models, fixed effects included killing method, bird type, bird order, and interactions between major factors. Co-variates included bird weight and stockworker experience. For both reflex data (264 birds) and post-mortem data (all birds), GLMMs were used to compare consistency across stockworkers. Model set-up was as described above for both types of measure, but stockworker was no longer included as a random effect and instead listed as a fixed effect.

## 3. Results

Mean (±SE) bird weights across the three bird types were: broiler = 2.38 ± 0.9 kg, layer hen = 2.8 ± 0.9 kg, and turkey = 12.7 ± 4.0 kg. Variation between the stockworker biometric measures was minimal and not significant. The means (±SE) for key stockworker parameters were; age = 40 ± 1 years, experience = 20 ± 1 years, height = 1.8 ± 0.5 m, and weight = 92.2 ± 0.6 kg.

A total of 2400 birds were killed within the trial. While every attempt was made to keep the kill rate within each session at two minutes per bird, due to unavoidable technical issues (e.g., CPK jamming or multiple kill attempts required) the rate was not always consistent (Table 1), and in some cases the kill rate within a session was slightly altered in order to compensate and maintain the total time of session to 3.5 h. The longest delays were seen in the CPK on the layer farm due to technical issues with the killing device.

### 3.1. Killing Performance

Both the CPK and cervical dislocation were highly successful, achieving over 96% kill success on the first attempt, with rapid death confirmation post application (CPK = 99.1% (1200 birds); cervical dislocation (manual + mechanically-assisted) = 97.3% (1200 birds), including manual = 96.9% (800 birds) and mechanically-assisted = 98.3% (400 birds)). There was no difference between the CPK and combined cervical dislocation methods in terms of kill success, or within cervical dislocation methods. There was no interaction between killing method and bird type. There was a significant interaction between kill success and kill sequence (*p* = 0.023), in that success improved as time went on within a session, with the CPK improving more than cervical dislocation methods (mean kill success rates for CPK: 98.4% (birds 1–20) to 99.6% (birds 80–100 cervical dislocation: 97.6% (birds 1–20) and 97.3% (birds 80–100)). There was no interaction between kill sequence and species. The main cause for kill success failures was multiple attempts (e.g., double pulls in cervical dislocation [17] or misfires in the CPK). The maximum number of attempts recorded was three, which were in the cervical dislocation methods for layers and turkeys (overall cervical dislocation mean (±SE) = 1.0 ± 0.2). For the CPK method, the maximum attempts recorded in all bird types was two (overall CPK mean (±SE) = 1.0 ± 0.1). There was no significant difference between number of kill attempts between all methods. There was no evidence that weight or stockworker had a significant effect on kill success, however, this may have been limited due to the low number of unsuccessful kills for comparison.

**Figure 2:**
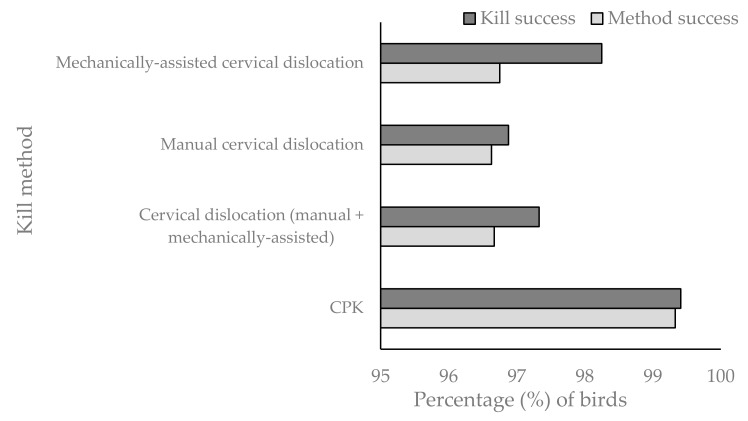
Bar chart providing a comparison between the percentage of birds successfully killed (kill success = single kill attempt resulting in rapid death) and the percentage of birds where the method application was optimal (method success = single kill attempt resulting in rapid death and optimal anatomical damage) for each kill method (CPK (*n* = 1200 birds), cervical dislocation (manual + mechanically-assisted) (*n* = 1200 birds), manual cervical dislocation alone (*n* = 800 birds), and mechanically-assisted cervical dislocation alone (*n* = 400 birds). Note that the Y axis scale starts at 95%.

In all killing treatments, method success was numerically lower than kill success, however, a minimal difference was seen with the CPK, suggesting that when it was successfully applied, it produced optimal damage to the bird (Figure 2). There was an interaction between kill method and kill sequence (*p* = 0.040), again suggesting that method success improved over time, but there was no effect of species or weight on method success.

### 3.2. Post-Mortem Parameters for Cervical Dislocation

**Figure 3:**
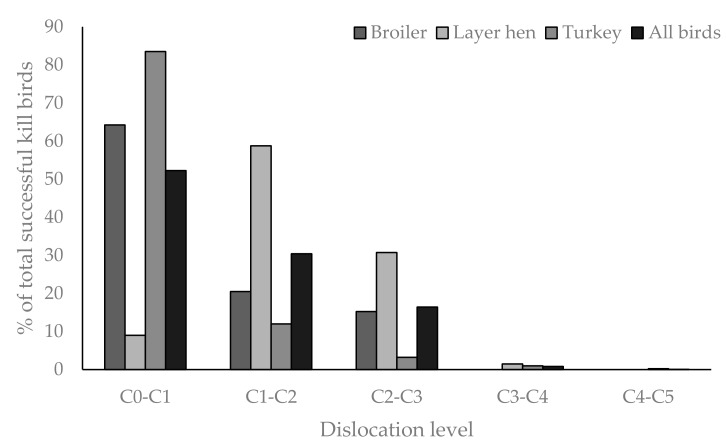
Bar chart showing the range of cervical dislocation levels achieved in successfully killed birds by cervical dislocation across all stockworkers, described as the proportion of birds (%) dependent on bird type (*p* < 0.0001).

For birds that were successfully killed by cervical dislocation (97.3%), the majority received a C0–C1 dislocation (53.3%), did not have the neck skin torn (97%), and only 24.2% of birds had one or more of the carotid arteries severed. Dislocation level had a significant effect on kill success (*p* = 0.042) and method success (*p* < 0.0001), with both kill and method success being more likely with a higher dislocation (kill success (y) C0–C1 = 53.3%, C1–C2 = 29.6%, C2–C3 = 16.3%, >C3–C4 = 0.8%; method success (y) C0–C1 = 53.7%, C1–C2 = 29.5%, C2–C3 = 16.3%, >C3–C4 = 0.5%). There was also a significant interaction between dislocation level and species (*p* < 0.0001), with 83.5% of turkeys receiving a C0–C1 dislocation, while only 52–65% of broilers and layers received the same. However, it is interesting to note that no broilers received a dislocation lower than C2–C3, while the lowest recorded dislocation for hens and turkeys was C4–C5 (Figure 3). Body weight had no effect on dislocation level or number of carotids severed. Whether one or more carotid arteries were severed was effected by bird type (*p* = 0.036), with artery severance more likely in turkeys (mean (±SE) = 1.2 ± 0.1 carotid arteries) compared to layer hens (0.1 ± 0.1), however, bird type was confounded with cervical dislocationmethod. Broilers were removed from analysis as no carotids were ever severed, but stretch damage was noted. Stockworker had an effect on the mean level of dislocation achieved (*p* = 0.002), with stockworker B and D both on average achieving a C0–C1 in >96% of their birds, while the joint worst performing stockworkers (E and G) achieved a C0–C1 in less than 20% of their birds. However, the stockworker effect was confounded with bird type: both stockworkers B and D were from the turkey farm, while stockworkers E and G were from the broiler farm. Kill sequence had no effect on dislocation level, carotid artery severance, and skin damage.

### 3.3. Post-Mortem Parameters for the Captive Bolt

For birds that were successfully killed by the CPK (99.1%), over 90% received damage to all regions of the brain assessed (forebrain (left (97.7%) and right (90.2%)), midbrain (left (99.2%) and right (99.2%)), and cerebellum (97%)). Statistical analysis could not be carried out on brain damage data because of its lack of variation and the low number of unsuccessful kills for comparison.

### 3.4. Reflex and Behaviour Parameters

**Figure 4:**
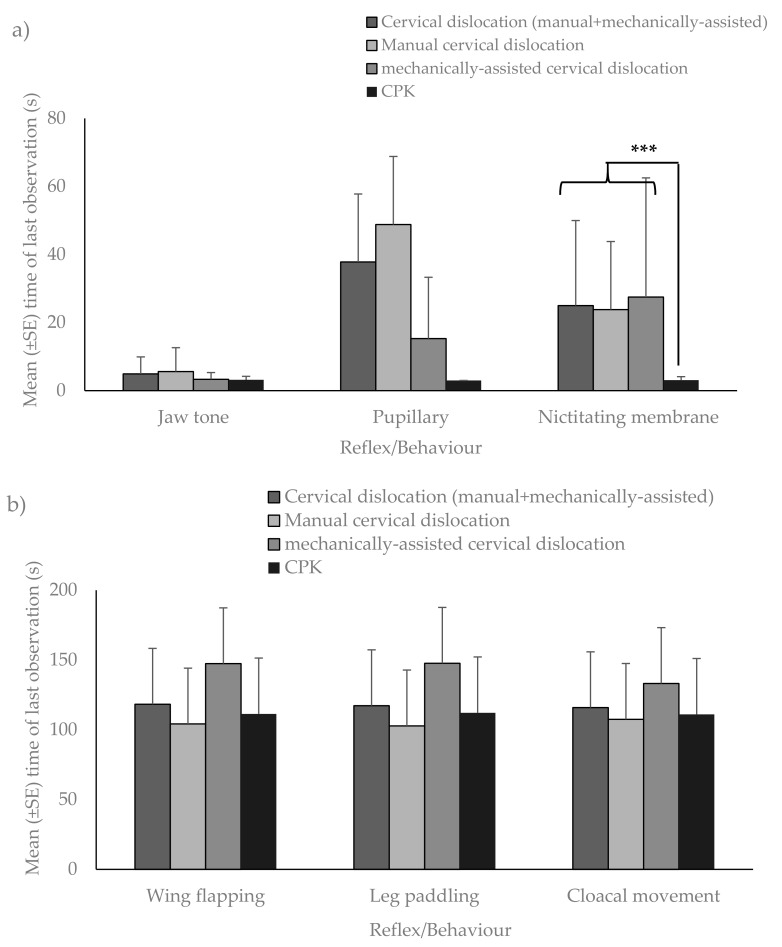
Comparison of mean (±SE) times of last observations for (**a**) jaw tone, pupillary, and nictitating membrane; and (**b**) wing flapping, leg paddling, and cloacal movement post successful method application for all killing methods. Asterisks (*) indicate that there was a significant difference between groups (*** = *p* < 0.001).

Of the birds whose reflexes were measured, no birds showed rhythmic breathing post successful method application for any method. In general, birds killed with CPK displayed fewer reflexes post successful method application than those killed by cervical dislocation (Table 2), and the data also suggest that mechanically-assisted cervical dislocation was better than the manual method at rapidly eliminating jaw tone, pupillary, and nictitating membrane reflexes (Figure 4). The CPK had the shortest mean times for all reflexes (significant only for nictitating membrane (*p* < 0.001); pupillary reflex could not be modelled as too few birds killed with the CPK showed this reflex). Jaw tone was abolished with all methods in a mean time of <6 s after method application, with cervical dislocation taking 1.7 s longer than CPK, and showing more between bird variation. There was no effect of kill sequence on any behaviour or reflex parameter.

The mean durations of reflexes and behaviours post killing method application for cervical dislocation (Table 3) and CPK (Table 4) are reported. With cervical dislocation, time to last observation of pupillary reflex (*p* < 0.001) and nictitating membrane reflex (*p* < 0.001) was reduced if one or more carotid arteries were severed. Dislocation level was also associated with time to loss of jaw tone (*p* = 0.009), pupillary (*p* = 0.003), and nictitating membrane (*p* < 0.001), with higher dislocations associated with more rapid loss of each reflex. Statistical analysis could not be carried out on reflexes in relation to brain damage data from CPK use because of its sparseness, lack of variation, or because reflexes were not seen.

## 4. Discussion

Practical evaluations of on-farm killing methods in commercial environments are vital to accurately assess performance across multiple operators and in continuous use. In this study, both the CPK and cervical dislocation were highly successful with no evidence that one approach was significantly better than the other, in all three bird types. The main cause for kill success failures was multiple attempts (e.g., double pulls in cervical dislocation methods [17] or misfires in the CPK).

Our standardization of kill rate and total kill session duration allowed all parameters to be measured (e.g., reflex and post-mortem measures), but also prevented a potential confound of some methods being faster to perform and potential fatigue effects. Thus, the experiment investigated the effects of repeated and sequential use of each method, which, while incorporating an element of fatigue, did not reflect maximal commercial killing rates with the methods. Nevertheless, there was no evidence of a reduction in kill success or method success with sequential use. In fact, the opposite was observed with some evidence of improved kill success with greater sequential use of the CPK, possibly reflecting within-session improvement with practice. This was not observed for cervical dislocation. However, the kill fail rate across all methods was very small (43/2400) and the *n* varied across killing methods, so this significant relationship shows only marginal evidence for this effect. The lack of improvement or reduction in performance for cervical dislocation could be attributed to the substantial experience of the stockworkers, limiting the scope for improvement, or likelihood of poor performance. There was also no evidence that sequential killing of certain species or bird weights were associated with decreased kill success or method success. The fixed killing rate employed may have masked fatigue by allowing recovery between kills, and some delays caused by technical issues may have also had an influence. However, it could be argued that the technical issues experienced would be at least as likely to occur with commercially-relevant killing rates.

The CPK was the most prone to technical issues, which resulted in the longest delays. Twice, the CPK jammed due to a build-up of carbon deposits; twice, delays were caused by mis-fires or, in one case, partial explosion of the cartridge. On three occasions, the CPK had to be swapped with the spare in order to continue the experiment (after 71, 65, and 12 consecutive shots). The CPKs were cleaned at the end of every day (i.e., after 100 shots) by a trained individual, however, this did not prevent these issues from occurring repeatedly. It was noted that the recuperating sleeves which return the bolt to its initial firing position suffered higher than expected degradation after approximately 600 shots, and this may have contributed to failures. It is also essential to retrieve all fired cartridges, which can be difficult and time consuming, particularly in commercial settings on deep-litter floors. Another practical issue with the CPK is the requirement for the bird to be restrained, in this case, it was by another stockworker, however, in a commercial setting, this is unlikely and instead a killing cone or a rope would be used to hang and shackle the bird by the legs. This highlights that it is a potentially less convenient method than manual cervical dislocation, which requires no additional tools or support to apply.

Although the mechanically-assisted cervical dislocation was a form of cervical dislocation, care should be taken (because of the techniques involved) when comparing the results from previous studies or grouping it with manual cervical dislocation. For this reason, we have reported the data with the two methods combined and separated. The primary issue identified with the dislocation methods is the variation in application between stockworkers, as seen in post-mortem measures of dislocation level which in turn was related to reflex responses. Variation in manual cervical dislocation performance across multiple operators has been observed previously [17]. This emphasizes the importance of training and its standardization in the poultry industry. In this study, kill success did not vary by bird type, although the stockworker data from the turkey farm were confounded with a different cervical dislocation method. Previous studies [16,17] comparing manual and a form of mechanically-assisted cervical dislocation showed the same higher success and reliability for manual cervical dislocation in broiler stockworkers compared to layer hen stockworkers, perhaps because broiler stockworkers more regularly kill birds with the method. It is also important to note that the exact manual technique used varied across the broiler and layer stockworkers, as documented in Martin et al. [17]. The majority (53.3%) of birds which were successfully killed by cervical dislocation methods received a C0–C1 dislocation. This focuses the anatomical damage to the top of the spinal cord and possibly the base of brain stem. Damage to this area is associated with spinal cord concussion, neurogenic shock, and loss of consciousness [31,32,33,34,35]. Results reporting the majority of dislocations as C0–C1 have been reported previously [15,16,17], suggesting that overall, cervical dislocation is consistent. Interestingly, the mechanically-assisted cervical dislocation was the most successful method in severing carotid arteries, suggesting that the force produced and resultant stretch by using the operator’s leg was greater than the use of the arm, however, the method was confounded with bird type (turkeys). Continued use of cervical dislocation methods did not change the anatomical trauma induced in the birds (e.g., dislocation level, carotid artery severance, and skin damage), suggesting that the EU legislation, which restricts the use of manual cervical dislocation to 70 birds per day [12], is highly conservative. This result was also seen with repeated application of the CPK, where continued use in 100 birds was not an issue in terms of welfare consequences. Unlike in previous studies where heavier birds were more difficult to dislocate at C0–C1 compared to lighter birds, in this study, cervical dislocation performance (kill success and trauma) was not affected by bird weight [16,17]. Bird weight had no effect on the performance of the CPK.

As with trauma, abolition of reflexes and durations of behaviours were not affected by repeated application of the methods, suggesting that for both methods, killing up to 100 birds resulted in reliable and rapid kills (at least at the rates used). Overall, the CPK was associated with better welfare outcomes, with shorter durations of reflex and behaviour persistence, and fewer birds exhibiting them. Similar results have been reported previously, showing captive bolt methods to be humane when applied successfully [7,14,24,25]. However, despite its welfare credentials, any long delays (e.g., due to technical problems) meant birds were restrained for excessive periods or even experienced a partial shot, which compromised welfare. It is also worth noting that, while the CPK produced a faster kill, the availability of a working and loaded device may be a barrier to its use on farm. Manual cervical dislocation may be employed without delay to end the life of a suffering bird (under 3 kg) that has been discovered by a stockworker, a benefit which may negate marginal reductions in time to loss of consciousness.

## 5. Conclusions

Both of the methods tested (CPK and cervical dislocation) are highly successful and acceptable on-farm culling methods for broilers, layers, and turkeys. Reflex and behaviour measures showed that both methods caused loss of brain function rapidly, however, there was more variation in birds killed by neck dislocation than CPK. High neck dislocation was associated with improved kill success and more rapid loss of reflexes compared to lower level dislocations for cervical dislocation. The CPK caused damage to multiple brain areas with little variation; post-mortem measures for neck dislocation showed greater variation as a result of the stockworker applying the technique. There was no evidence of a negative effect of sequential application of the methods on killing efficacy or welfare impact in this experiment, up to 100 birds at a killing rate of one bird per 2 min. However, it is important to note that technical difficulties with the CPK resulted in excessive delays, which could have compromised bird welfare in a realistic commercial setting. 

## Figures and Tables

**Table 1:** Descriptive statistics (mean, SE, SD, minimum, and maximum) for the time gap between kills within sessions for each kill method. CPK = Cash Poultry Killer (CPK).

Method	Bird Type	Time Gap Between Kills within Session (min)				
		Mean	SE	SD	Min	Max
Manual cervical dislocation	All	2.00	0.00	0.09	1	4
	Broiler	2.00	0.00	0.00	2	2
	Layer	2.00	0.01	0.12	1	4
	Turkey	2.00	0.00	0.09	1	3
CPK	All	2.00	0.00	0.28	1	11
	Broiler	2.00	0.00	0.00	2	2
	Layer	2.00	0.01	0.47	1	11
	Turkey	2.01	0.00	0.13	1	4

**Table 2:** Proportion (%) of birds displaying reflexes/behaviours post successful method application at any time for both cervical dislocation methods (manual cervical dislocation and mechanically-assisted cervical dislocation) and the captive bolt (CPK).

Reflex/Behaviour	Manual Cervical Dislocation	Mechanically-Assisted Cervical Dislocation	CPK
Jaw tone	18.6	4.8	3.1
Pupillary	100.0	35.7	0.0
Nictitating	80.2	50.0	1.1
Rhythmic breathing	0.0	0.0	0.0
Wing flapping	100.0	100.0	100.0
Leg paddling	100.0	100.0	100.0
Vent movement	95.3	100.0	95.4

**Table 3:** Mean (±SD) duration (s) of reflexes and behaviours post cervical dislocation (manual + mechanically-assisted) killing method application in relation to achieved (Y/N) post-mortem parameters.

Cervical Dislocation Post-Mortem Parameters		Reflex/Behaviour Duration (s)					
		Jaw Tone	Pupillary	Nictitating Membrane	Wing Flapping	Leg Paddling	Cloacal Movement
neck skin intact	N	3.0 ± 0.0	3.0 ± 0.0	37.7 ± 40.6	143.8 ± 53.9	143.8 ± 53.9	140.0 ± 50.5
	Y	4.9 ± 5.8	38.9 ± 24.5	26.0 ± 31.0	118.4 ± 44.0	117.4 ± 43.7	114.1 ± 43.4
≥1 carotid arteries severed	N	5.3 ± 6.5	45.7 ± 20.8	28.9 ± 29.3	113.2 ± 41.8	112.0 ± 41.2	111.4 ± 41.5
	Y	3.7 ± 2.1	13.2 ± 20.2	18.3 ± 35.8	138.5 ± 47.2	138.5 ± 47.2	125.9 ± 48.9
dislocation level >C0–C1	N	3.6 ± 2.6	26.3 ± 23.9	17.1 ± 24.8	115.1 ± 47.2	115.9 ± 46.5	115.3 ± 38.9
	Y	5.8 ± 7.0	45.8 ± 22.4	32.7 ± 33.7	121.9 ± 42.3	119.8 ± 42.4	114.7 ± 46.9
dislocation level >C1–C2	N	4.6 ± 5.3	36.3 ± 24.7	21.1 ± 24.5	116.3 ± 43.7	115.1 ± 43.2	113.4 ± 40.9
	Y	6.2 ± 7.7	45.3 ± 24.6	52.5 ± 45.9	133.2 ± 45.5	133.9 ± 45.7	122.8 ± 55.8

**Table 4:** Mean (±SD) duration (s) of reflexes and behaviours post CPK method application in relation to achieved (Y/N) post-mortem parameters.

CPK Method Post-Mortem Parameters		Reflex/Behaviour Duration (s)					
		Jaw tone	Pupillary	Nictitating Membrane	Wing Flapping	Leg Paddling	Cloacal Movement
scalp skin intact	N	3.2 ±1.2	3.0 ± 0.0	3.1 ± 0.8	112.1 ± 50.6	112.3 ± 48.5	110.6 ± 48.8
	Y	3.2 ± 1.2	3.0 ± 0.0	3.0 ± 0.0	106.7 ± 57.0	109.1 ± 55.5	110.9 ± 47.9
left forebrain damaged	N	3.0 ± 0.0	3.0 ± 0.0	10.0 ± 0.0	130.0 ± 60.6	125.0 ± 60.0	135.0 ± 52.7
	Y	3.2 ± 1.2	3.0 ± 0.0	3.0 ± 0.0	110.4 ± 52.0	111.2 ± 50.1	110.1 ± 48.3
right forebrain damaged	N	3.0 ± 0.0	3.0 ± 0.0	3.0 ± 0.0	105.4 ± 48.0	104.2 ± 47.3	111.0 ± 53.9
	Y	3.2 ± 1.3	3.0 ± 0.0	3.1 ± 0.8	111.4 ± 52.7	112.3 ± 50.5	110.7 ± 48.0
left midbrain damaged	N	3.0 ± 0.0	3.0 ± 0.0	3.0 ± 0.0	125.0 ± 0.0	125.0 ± 0.0	125.0 ± 0.0
	Y	3.2 ± 1.2	3.0 ± 0.0	3.1 ± 0.7	110.7 ± 52.3	111.4 ± 50.3	110.6 ± 48.5
right midbrain damaged	N	3.0 ± 0.0	3.0 ± 0.0	3.0 ± 0.0	170.0 ± 0.0	170.0 ± 0.0	170.0 ± 0.0
	Y	3.2 ± 1.2	3.0 ± 0.0	3.1 ± 0.7	110.0 ± 52.0	111.0 ± 50.0	110.3 ± 48.3
brain stem damaged	N	3.0 ± 0.0	3.0 ± 0.0	3.0 ± 0.0	145.0 ± 0.0	145.0 ± 0.0	145.0 ± 0.0
	Y	3.2 ± 1.2	3.0 ± 0.0	3.1 ± 0.7	110.0 ± 51.7	110.7 ± 49.6	110.0 ± 48.3
cerebellum damaged	N	3.0 ± 0.0	3.0 ± 0.0	3.0 ± 0.0	76.3 ± 62.9	91.3 ± 53.9	53.3 ± 34.2
	Y	3.2 ± 1.2	3.0 ± 0.0	3.1 ± 0.7	111.9 ± 51.6	112.1 ± 50.1	112.5 ± 47.7
