# Erratum: Martin, J.E., et al. Welfare Risks of Repeated Application of On-Farm Killing Methods for Poultry. *Animals* 2018, *8*, 39

**DOI:** 10.3390/ani8060094

**Published:** 2018-06-11

**Authors:** Jessica E. Martin, Dale A. Sandercock, Victoria Sandilands, Julian Sparrey, Laurence Baker, Nick H. C. Sparks, Dorothy E. F. McKeegan

**Affiliations:** 1 The Royal (Dick) School of Veterinary Studies and The Roslin Institute, Easter Bush Campus, The University of Edinburgh, Edinburgh EH25 9RG, UK; Nick.Sparks@roslin.ed.ac.uk; 2 Animal and Veterinary Science Research Group, Scotland’s Rural College (SRUC), West Mains Road, Edinburgh EH16 4SA, UK; dale.sandercock@sruc.ac.uk (D.A.S.); Vicky.Sandilands@sruc.ac.uk (V.S.); laurence.baker@sruc.ac.uk (L.B.); 3 Livetec Systems Ltd., Building 52, Wrest Park, Silsoe, Bedford MK45 4HS, UK; sparrey@livetecsystems.co.uk; 4 Institute of Biodiversity, Animal Health and Comparative Medicine, College of Medical, Veterinary & Life Sciences, University of Glasgow, Glasgow G61 1QH, UK; Dorothy.McKeegan@glasgow.ac.uk

The authors wish to make the following correction to their paper [1].

In the Materials and Method Section, “All stockworkers completed a full training day in the CPK, provided and audited by the HSA in November 2012, and all were deemed competent by the HSA representative” should read as “All stockworkers completed a full training day in the CPK, provided by the HSA in October and November 2012.”

The authors would like to apologize for any inconvenience caused. The change does not affect the scientific results. The manuscript will be updated and the original will remain online on the article webpage.
